# Neuromas at the castration site in geldings

**DOI:** 10.1186/s13028-019-0479-8

**Published:** 2019-09-24

**Authors:** Emma Angelina Bengtsdotter, Stina Ekman, Pia Haubro Andersen

**Affiliations:** 1 Department of Biomedicine and Veterinary Public Health, Swedish University of Agricultural Sciences, 750 07 Uppsala, Sweden; 2 Department of Clinical Sciences, Swedish University of Agricultural Sciences, 750 07 Uppsala, Sweden

**Keywords:** Castration, Gelding, Horse, Neuroma, Pain

## Abstract

**Background:**

Inguinal pain, unexplained hind limb lameness, back pain or behavioural problems in geldings could be attributable to painful neuromas that develop as a consequence of crushing and severing the testicular nerves during castration. The presence of neuroma in this anatomical location has never been reported, hence the knowledge of possible clinical relevance is limited. The aim of this study was to histologically investigate the testicular nerves at the castration site in geldings for the presence of neuromas. Proximal spermatic cord remnants were collected from 20 geldings admitted to routine post mortem examination for various reasons. The time of castration was unknown, but it had not been performed during the last year. Spermatic cord specimens were immersed in 10% formalin, trimmed, dehydrated, embedded in paraffin, sectioned and stained with haematoxylin and eosin (HE) for light microscopy. Identification of nerve tissue was done by immuno-localization of nerve specific enolase (NSE).

**Results:**

Neuromas were found in 21 spermatic cords from 13 geldings and were bilateral in eight of the horses. The neuromas consisted of areas with small groups of non-neoplastic proliferations of peripheral neural tissue. The tissue included neurofilaments and Schwann cells, intermingled or surrounded with, epineural, perineural and endoneural fibrous tissue. The neural tissue immunostained positive with NSE.

**Conclusions:**

This study showed neuromas of the remnant testicular nerves at the site of castration. Further studies are required to establish if these neuromas in the castration site are painful and if certain castration methods promote their formation. Future studies should also investigate the clinical consequence of these neuromas for the individual horse.

## Background

Worldwide, castration is the most commonly performed surgery in horses. In Sweden, the majority of stallions are castrated in the field at the age of 1–2 years, either during a standing procedure, or during lateral recumbency induced by short acting intravenous anesthetics. Most of these castrations are performed as semi-closed castrations, where testes are removed through scrotal skin and parietal vaginal tunic incisions. Hemostasis is achieved by crushing of the cord located in the vaginal tunic without use of ligatures. Scrotal wounds are left open for drainage. A minority of horses are castrated at hospitals, using inguinal or scrotal approaches, closed castration and application of ligature for hemostasis and wound closure for primary healing [1].

Castration is associated with postoperative complications such as swelling/oedema, haemorrhage, infection, septic funiculitis of the stump of the cord eventually resulting in ascending peritonitis, omental herniation and eventration [1, 2]. While these acute or short-term complications are rather well-investigated, late complications are less studied. Horse owners may present geldings with rather vague signs occurring after the castration, such as changed behaviour, gait and urination habits or decreased riding performance. While there are few studies on these problems, it is generally accepted that soreness related to the post-operative scar may be as a source of gait modification [1]. Treatment includes surgical removal of massive scarring tissues, including larger or smaller areas of fibrous tissues, at the castration site. But no research has been performed on the outcome of the surgery or the possible pathology involved.

The equine testis is innervated by a nerve plexus of autonomic sympathetic nerve fibres. These lead from the aortic plexus to the caudal mesenteric plexus and from the splanchnic lumbar nerve, accompanying the testicular vessels in the spermatic cord [3]. From a pathological perspective, all castration techniques are associated with crushing or compression of the spermatic cord tissues. The most common surgical approaches additionally involve cutting, tearing or crushing of peripheral nerves of the skin, muscle and subcutaneous tissues. The nerves severed by the castration comprise the testicular nerves of the spermatic cord, and branches of peripheral nerves such as those of the somatic genitofemoral, ilioinguinal and pudenda nerves that innervate the cremaster muscle, scrotal skin and vaginal tunic [4].

When a nerve is severed, the proximal part reacts by sprouting multiple axons [5], to connect with the distal part of the nerve. These sprouting fibres proliferate into the extra-endoneural environment in a disorganized way, if the endoneurium is damaged with the distal nerve missing. This can also happen due to a too great distance between the proximal and the distal nerve ends or if the way is blocked by granulation tissue. These scenarios can occur after castration. A neuroma can result from the bundles of disorganized nerve fibres and Schwann cells mixed together with fibroblasts, collagen, capillaries and myofibroblasts [5]. The neuroma is often surrounded by fibrous tissue proliferating from the perineurium, and the size of it depends on the axonal activity and amount of fibroblasts and Schwann cells. Most human neuromas are asymptomatic [6], but can become painful if chronically irritated, or if the free nerve endings of the neuroma are constantly stimulated.

Neuromas after castration have never been demonstrated, in either animals or humans. Interestingly, neuromas caused by surgical trauma to peripheral nerves are described after tail docking in pigs [7–9], lambs [10] and dogs [11], after neurectomy in horses [12, 13] and beak trimming in poultry [14]. Neuromas are also described in shoulder areas of sows with deep ulcerations [15]. In human medicine, neuromas following amputation may cause chronic or neuropathic pain [16, 17]. In veterinary medicine, aversive reactions to touching or palpating the area of neuromas are described only briefly in some of the above cited literature [11, 12, 15]. This is probably due to the lack of validated methods for assessing chronic or neuropathic pain in animals.

On the basis of this, we hypothesized that amputation neuroma may develop at the castration site in horses, and that some of these would produce chronic or neuropathic pain. The aim of this study was to detect the presence of neuromas involving the testicular nerves more than 1 year after castration. This was done by histological investigation of the healed castration site of the spermatic cord.

## Methods

### Animals

The study used necropsy material from 20 geldings of different breeds and ages, euthanized for reasons unrelated to the urogenital system. The geldings were undergoing routine postmortem examination at the Swedish University of Agricultural Sciences (SLU). As a control, necropsy material (spermatic cord and testicular nerves) was collected from a stallion (Icelandic breed, age 3 years). The castration histories of the 20 geldings were not known, but the castration had not been performed during the previous year.

### Dissection of the castration site

With the horse in dorsal recumbence, the inguinal tissue was dissected from the area of the external inguinal ring and towards the castration scar in both sides, to reveal the amputation site of the spermatic cord. The cord was grossly inspected for appearance in situ. The remaining right and left spermatic cords and attached surrounding tissues were dissected and removed in toto. All the resected tissues were immersed in 10% neutral buffered formalin for 24 h at one to 48 h after euthanasia. The length of the specimens was subjectively classified as short (< 5 cm), medium (5–10 cm) or long (> 10 cm).

### Histology

After fixation, the cords were trimmed from surrounding fat tissue and the castration site divided in proximal-, mid- and distal segments and arranged longitudinally (n = 3/cord). Tissue samples from the right spermatic cord of the stallion were divided as cross-sections in proximal- and mid-segments and as a longitudinal section of the mid-segment (n = 3). All trimmed samples were dehydrated, embedded in paraffin, and cut into 4-μm sections. One tissue section from each segment were stained with haematoxylin and eosin (HE) and examined by light microscopy.

**Fig. 1:**
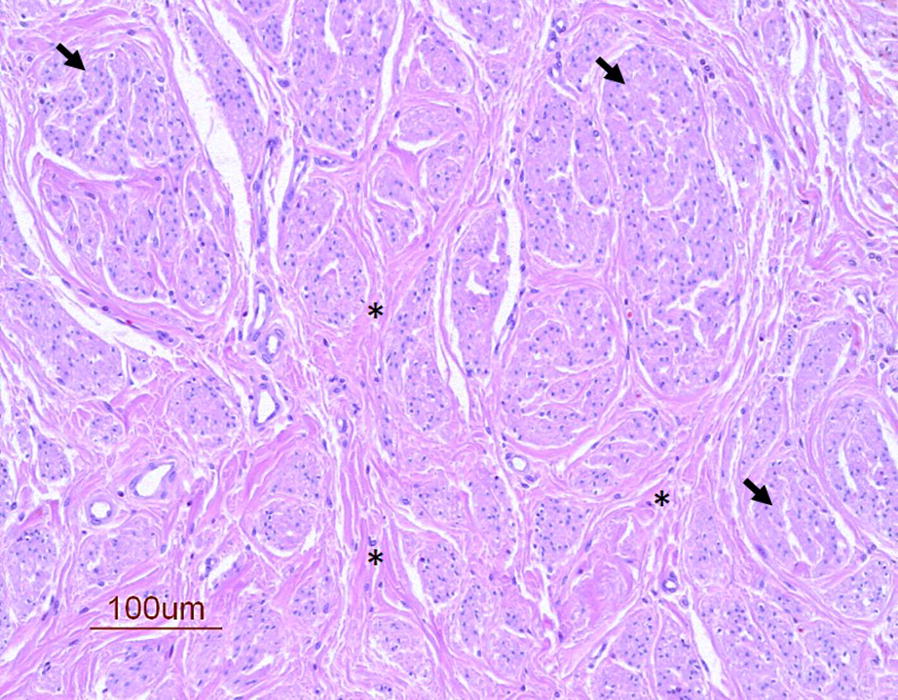
Typical neuroma. Neuroma stained with HE from a gelding (no. 12). Neural proliferation of variable sized micro-fascicles of axonal sprouts (arrows) in fibrous connective tissue (asterisk)

Neuromas were histologically defined as an abnormal appearance or irregular distribution of nervous tissue in which there were small groups of non-neoplastic proliferations of peripheral neural tissue. The size of the neuromas was not measured. The tissue included neurofilaments and Schwann cells, intermingled or surrounded with epineural, perineural and endoneural fibrous tissue (Fig. 1).

### Immunohistochemistry

Sections with putative neuromas identified by histology were selected for further immunohistochemical characterisation of the peripheral nerve tissue morphology. These selected slides were labelled with mouse monoclonal IgG_1_ kappa antibodies (diluted 1:100 and 1:1000) against human nerve specific enolase (NSE)^1^ for visualisation of neuronal cells [18]. An indirect method with a horseradish peroxidase–labelled polymer (HRP) conjugated to the secondary antibody^2^ was used to visualise the staining by light microscopy. As negative control, an isotype of a mouse IgG,^3^ diluted to the same protein concertation as the primary antibody, was used. The testicular nerves from the spermatic cord of the stallion were used as positive control.

In brief, the sections were deparaffinised, then rehydrated in descending grades of ethanol and washed in phosphate-buffered saline (PBS).^4^ Antigen retrieval was done using Na-citrate buffer (pH 6.0) for 20 min at 92 °C in a floatation bath. To quench the endogenous peroxidase activity, 3% hydrogen peroxide was used prior to incubation with the primary antibody or the mouse IgG for 30 min at room temperature. A secondary antibody labelled with polymer HRP anti-rabbit/mouse was added for 30 min and a 3,3′-diaminobenzidine (DAB)-substrate buffer for visualisation (for protocol see Additional file 1).

## Results

### Gross findings

The horses ranged in age from 6 to 25 years. Breed and reason for euthanasia are given in Table 1. Many were colic subjects presented with acute peritonitis, but none had adhesions that interfered with the castration site. By gross inspection, the spermatic cords differed in length, thickness and consistency. The length from the external inguinal ring to the distal end of the proximal stump varied, from 3 to 20 cm. The thickness and consistency of the cord was thin, soft and whitish without any visible structures or abnormalities in some horses and thick and firm with visible vessels and fibrous structures in others. Length classification is given in Table 1. In one of the horses (no. 11), the right spermatic cord could not be localised. Swellings possibly being neuromas were searched for grossly but were not identified in any spermatic cords.

**Table 1:** Age, breed, subjective length of spermatic cord stump, presence of neuroma and reason for euthanasia of 20 geldings

Gelding	Age (years)	Breed	Length of stump	Neuroma left/right	Cause for euthanasia
1	25	Lusitano	Short	+/+	Heart failure
2	7	Quarter horse	Medium	−/−	Colic
3	7	Ardennes horse	Long	−/+	Chronic lameness
4	6	SWB	Medium	+/−	Back problem
5	8	Standard breed trotter	Short	−/−	Chronic illness
6	25	Welsh Cob.	Long	+/+	Tumour
7	5	North Swedish draft horse	Medium	−/+	Lameness
8	10	Cross breed	Long	−/+	Ventricular dilatation
9	11	Warmblood	Medium	−/−	Colic
10	5	Standard breed trotter	Medium	+/+	Neurological signs
11	12	Coldblooded trotter	Short	−/na	Colic
12	15	Anglo arab	Long	+/+	Ventricular tumor
13	17	Icelandic horse	Long	+/+	Sudden death
14	12	Friesian	Medium	−/−	Colic
15	10	Arab horse	Medium	−/+	Colic
16	7	PRE	Short	+/+	Lameness
17	7	SWB	Long	−/−	Colic
18	22	SWB	Medium	+/+	Colic
19	8	Icelandic horse	Long	−/−	Colitis
20	18	Swedish pony	Medium	+/+	Colic

Presence (+) or absence (−) of neuroma

*na* not analysed, *SWH* Swedish Warmblood Horse, *PRE* pura raza española

### Histology including immunohistochemistry

**Fig. 2:**
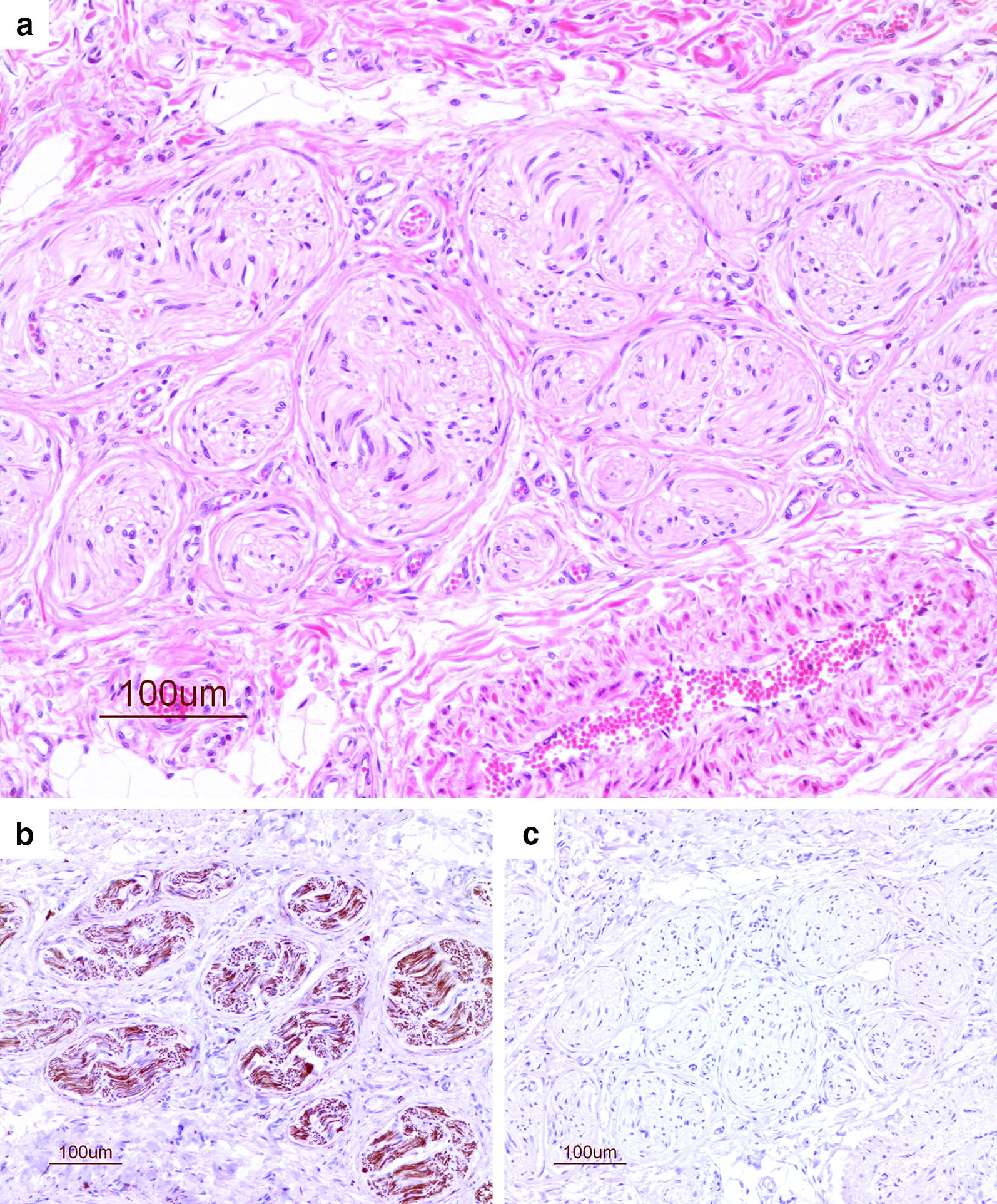
Normal nerves from the spermatic cord of a stallion. Nerves from the spermatic cord of a 3-year-old stallion. **a** Regular parallel bundles of neural tissue (HE), identified by **b** brown immuno-staining against NSE. **c** Negative control, stained with non-specific IgG shows no staining

A total of 120 sections were examined histologically from 40 spermatic cords (stallion included) and sections with putative neuromas were examined with immunohistochemistry (n = 31). The histologic structures of the spermatic cord from the stallion contained blood vessels comprised of arteries with thick walls, and the pampiniform plexus of veins. Adipose tissue and connective tissue were mingled between the veins; connective tissue consisted of fibroblasts and extracellular matrix, mainly collagen. The spermatic cord included multiple peripheral nerves, individually or in larger bundles, and the ductus deferens comprised a thick smooth muscle wall including arterioles, small veins and nerves. The mucosa of the duct was folded and lined by a pseudostratified epithelium. The nerve structures in the spermatic cord of the stallion were well organised, with parallel neurofilaments, of different sizes, surrounded by Schwann cells and myelin sheaths (Fig. 2a). These were clearly visible in the HE-stained sections and the nerve fibres stained positive for NSE (Fig. 2b). The wall of the ductus deferens displayed diffuse expression of NSE, due to its rich innervation of nerve plexus in the smooth muscle layer [19]. The sections immunostained with non-specific mouse IgG did not show any staining (Fig. 2c).

**Fig. 3:**
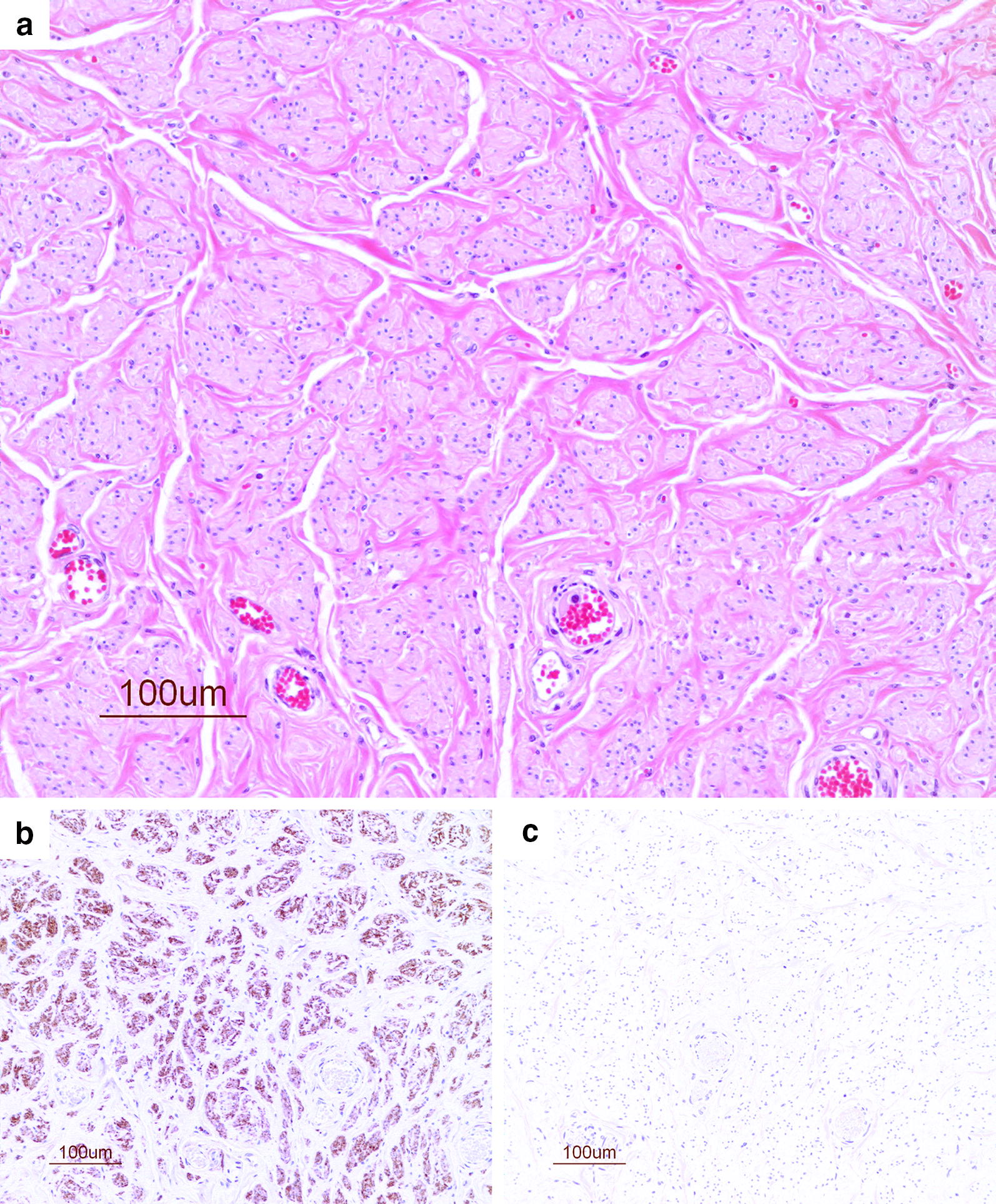
Neuroma from gelding (no. 13). **a** Irregular axonal proliferation of variably-sized micro-fascicles, in fibrous connective tissue (HE). **b** The same neuroma with axonal sprouts immunostained with antibodies against NSE (brown color). **c** The control incubated with nonspecific IgG, showing no staining

**Fig. 4:**
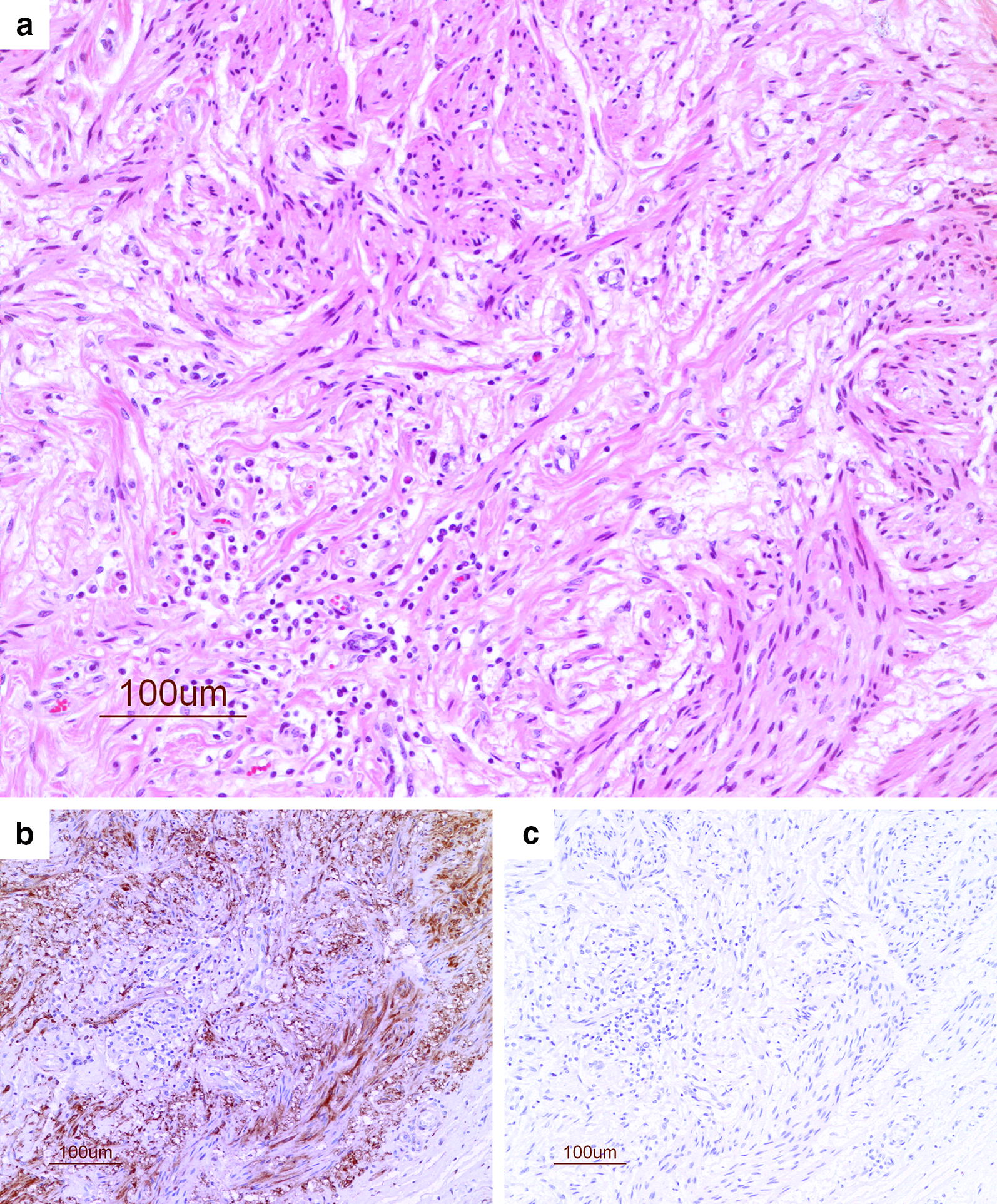
Neuroma from gelding (no. 15). **a** Irregular neural proliferation of variable-sized micro-fascicles with axonal sprouts in fibrous connective tissue (HE). An area infiltrated with inflammatory cells (neutrophils, lymphocytes and macrophages). **b** The axonal sprouts identified by immuno-staining with antibodies against NSE (brown color). **c** These show no staining when incubated with the non-specific IgG

The diagnosis of neuromas was made mainly on the basis of histological features found in the HE-stained sections. These showed non-neoplastic proliferation of an irregular fibrous, mature connective tissue with different-sized micro-fascicles of axonal sprouts. The neural origin of the micro-fascicles was confirmed by immunolabelling with NSE (Figs. 3, 4).

Neuromas (Figs. 3, 4) were found in 21 of 39 spermatic cords belonging to 13 of the 20 investigated geldings; 8 horses had bilateral neuromas (Table 1). The remnants of the spermatic cords of the geldings were often dominated by a large amount of fibrous, collagen-rich connective and adipose tissues. The amount and size of blood vessels varied between horses. Intact foci of small, peripheral nerve structures were seen in the adjacent tissue of some of the spermatic cords. A few horses (n = 3) had a mild multifocal inflammation, comprising neutrophils, lymphocytes and macrophages, with single hemosiderin laden macrophages within or near the neuroma.

## Discussion

This paper documents, for the first time, the occurrence of neuromas in connection with remnants of the spermatic cord after castration in horses. Neuromas were found in 54% of the spermatic cords, belonging to 65% of the 20 geldings. These neuromas were bilateral in 40% of the horses (Table 1).

Antibodies against NSE were used to identify the nerve tissue present in the irregular scar tissue of the remnants of the spermatic cords. NSE is a marker for nerve fibres [18], but is also found in neoplastic cells of neural and non-neural origin [20]. However it is generally accepted that NSE is a specific marker for neural and neuroendocrine tissue [21, 22].

The neuromas found at the castration site had histological features compatible with traumatic neuromas described after tail docking in pigs [9] where peri- and epineural connective tissue attenuates around proliferating axons. This has been suggested to be a mechanism to protect the neural fibers from injury due to contraction, which can cause pain after amputation. In the pig neuromas [9], S-100 was used showing presence of Schwann cells, however we confirmed axonal origin with NSE.

The gross appearance of the spermatic cord remnants displayed a large diversity in size and the amount of connective tissue and the gross examination was not standardised. The length of the remnants of the spermatic cord did not appear to be correlated with presence of neuromas. After neurectomy of horses [12] neuromas have been described grossly, however it is not clarified how the suspected neuromas were differentiated macroscopically from granulation tissue. In addition, these neuromas were found on large peripheral nerves, in contrast to the neuromas described in this study, which were found in the plexus innervating the testis.

Information about the horse’s age at castration or any post-surgical complications was not available, hence, it was not possible to correlate the neuromas with age at castration or castration method. However, as previously described, most castration techniques in Sweden include induction of haemostasis with an emasculator and/or a ligature on the spermatic cord and most horses are castrated before they are 2 years old. If this is applicable to the sample of horses in this study, the neuromas were present years after castration.

Neuromas are more likely to form in traumatised and inflamed tissues [23]. Most equine traditional castration methods include severe damage of the nerves of the spermatic cord and inflammation or infection is one of the most common complications [2]. In horses, most castration wounds are therefore left to secondary healing to eliminate inflammatory products from the crushed cords and tissues. Further, the inguinal area is a highly mobile region. From human research it is known that inflammation and movement are factors in development of painful neuromas (for review see [24]). Consequently, if this also applies to horses, the current methods for castration may need revision. In cases where clinical signs can be referred to the inguinal region, by palpation and local analgesia, presence of neuroma can be suspected.

Exactly why a neuroma becomes painful is not yet fully understood. In traumatic neuromas, afferent nerve fibres have been found, with a low conduction velocity and spontaneous electrical hyperexcitability [16, 25]. Changes in features or dispersion of potassium and sodium ion-channels can lead to ectopic activity of the axons, causing abnormal discharge patterns and thereby pain or paraesthesia [26, 27]. Mechanical stimulation of neuromas activates different responses of the afferent axons leading to hyperalgesia [16, 28]. Contraction of the wound can compress axons in the neuroma and is conjectured to be a trigger of pain or abnormal sensation [23]. It is not unlikely that at least some of the neuromas formed will be painful, given the anatomical location of the severed cords in a highly mobile area, and the high risk of formation of adherences due to infection.

The present study was not, however, designed to answer whether the neuromas were painful. Studies on human finger amputations report that 7.3% of the traumatic neuromas are symptomatic (caused pain or altered sensation) [6] and neuromas after inguinal hernia repair are often associated with chronic pain [29]. Although the peripheral nerves differ from the testicular ones in sensory and motor composition, the mechanisms responsible for development of painful neuromas could be similar. Future research should aim at classifying the neuromas as painful or non-painful. This could be done both clinically and with biomarkers for pain. The biomarkers could be used in vivo and measured in equine serum or by immunohistochemistry on histologic sections from the castration site. The nerve growth factor receptors are suggested markers with a high expression in the neuromas of patients reporting chronic neuroma pain [30]. An increased expression of substance-P has also been reported in painful neuromas compared to intact nerves [31]. Finally, alpha smooth muscle actin have shown a higher expression in painful neuromas compared to non-painful neuromas in humans [32].

Currently there are no validated methods for clinical investigation of horses with suspected painful castration neuromas. It is of great importance that such methods be developed. The clinical implications of possible chronic pain long after castration affect the welfare of the horse.

## Conclusions

This study is the first to document neuroma formation involving the testicular nerves in the remnants of spermatic cords at the castration site in geldings. If some of these neuromas are painful, they may explain pain in the inguinal area, unexplained hind limb lameness, lack of hind limb activation, behavioural problems, and unwillingness to perform certain movements.

## Supplementary information

**Additional file 1.** Protocol for immunohistochemistry of nerve specific enolase (NSE).

## Data Availability

The datasets used and/or analysed during the current study are available from the corresponding author on reasonable request.

## Footnotes

1 NSE, mouse monoclonal, Dako M0873, 367 mg/L.

2 Dako EnVision+ System-HRP anti-rabbit/mouse, K5007, Agilent technologies Santa Clara, USA.

3 Mouse IgG, Dako X0931.

4 PBS, 0.01 M, pH 7.4. Medicago AB, Uppsala, Sweden.
