# Supplementary material for: Neuromas at the castration site in geldings
Source: Acta Vet Scand. 2019 Sep 24;61:43. doi: 10.1186/s13028-019-0479-8 (PMC6760059; doi:10.1186/s13028-019-0479-8)
Supplement: Supplementary file 1 — Additional file 1. Protocol for immunohistochemistry of nerve specific enolase (NSE). [file 13028_2019_479_MOESM1_ESM.pdf]

Additional file 1. Protocol for immunohistochemistry of nerve specific enolase (NSE).

1. Incubate in 60°C for 20 minutes, cool down to approximately 37°C.
2. Deparaffinisation in Histo Lab Clear
    a. 15 minutes in 37°C
    b. 15 minutes in room temperature

3. Hydration performed in hood
    a. Ethanol 99%, 2 x 5 minutes
    b. Ethanol 95%, 1 x 5 minutes
    c. Ethanol 70%, 1 x 5 minutes

4. Rinse with de-ionized water 3 x 3 minutes
5. Incubate the slides in Na-citratebuffer (pH6) at 96°C for 20 minutes, cool down in room temperature for 20 minutes.
6. Rinse with de-ionized water 3 x 3 minutes

7. Quench endogen peroxidase with fresh 3% hydrogenperoxide (5 ml 30% $H2O2$ + 45 ml de-ionized water), 5 minutes in dark.
8. Rinse with PBS (Phosphate Buffered Saline) 3 x 3 minutes, mark with PAP-pen

9. Add primary antibody diluted in PBS. Add negative control diluted in PBS (see dilutions for each label underneath).
10. Incubate for 30 minutes at room temperature in humidity chamber.
11. Rinse with PBS 3 x 3 minutes

12. Add secondary antibody, labelled polymer HRP anti- rabbit/mouse.
13. Incubate for 30 minutes at room temperature in humidity chamber.
14. Rinse with PBS 3 x 3 minutes

15. Incubate with DAB+substrate buffer, check under microscope.
16. Rinse in water for 15 minutes.

17. Stain with Mayers Htx for 1 minute 30 seconds.
18. Rinse in water for 15 minutes.
19. Dehydration performed in hood
    a. Ethanol 70%, 1 x 1 minute
    b. Ethanol 95%, 1 x 1 minute
    c. Ethanol 99 %, 2 x 1 minute
    d. Ethanol 99 %, 1 x 5 minutes

20. Place in Xylene, 2 x 1 minute, then let it stand in Xylene for 15 minutes.
21. Mount

Primary antibody: NSE, mouse monoclonal, Dako M0873, 367 mg/l. Diluted 1:100.
Negative control: Neg Control Mouse IgG, Dako X0931, 100 mg/l. Diluted 3,5:100.

Using: Dako EnVision+ system, HRP anti-rabbit/mouse, K5007.
